# Estrogen hormone replacement therapy in incidental intracranial meningioma: a growth-rate analysis

**DOI:** 10.1038/s41598-020-74344-x

**Published:** 2020-10-21

**Authors:** Laura Dresser, Carlen Amy Yuen, Andrew Wilmington, Matthew Walker, Tilley Jenkins Vogel, Ryan T. Merrell, David Olayinka Kamson

**Affiliations:** 1 Department of Neurology, University of Chicago, Chicago, IL USA; 2 Pritzker School of Medicine, University of Chicago, Chicago, IL USA; 3 Department of Radiology, NorthShore University Health System, Evanston, IL USA; 4 Department of Obstetrics and Gynecology, NorthShore University Health System, Evanston, IL USA; 5 Department of Neurology, NorthShore University Health System, Evanston, IL USA; 6 Department of Neuro-Oncology, Sidney Kimmel Comprehensive Cancer Center at Johns Hopkins University, Baltimore, MD USA; 7 Neuro-Oncology Branch, National Cancer Institute, National Institutes of Health, Bethesda, MD USA

**Keywords:** CNS cancer, Neurology

## Abstract

Incidental meningiomas (IMs) are the most common intracranial neoplasms, especially in perimenopausal women. There is ongoing debate on whether their incidence is increased by hormone replacement therapy. Meningiomas often express estrogen receptors, which were linked to higher proliferative activity according to some reports. Consequently, there is a theoretical risk of estrogen-based HRT (e-HRT) leading to an increase in tumor growth and thus altering the natural history of IMs. However, clinical data is lacking to support this notion. To identify differences in the natural history of IM after e-HRT exposure. We queried the NorthShore Meningioma Database for patients with ≥ 6 months of e-HRT. They were compared with age-matched IM controls. Forty patients were included in the e-HRT group (mean age 62.1 ± 12.0 years; mean duration of HRT 5.3 ± 4.5 years) and 80 in the no-HRT group (mean age 62.2 ± 12 years). Radiographic appearance was similar between groups. The average 2D tumor diameter was 35% lower in the e-HRT group (*p* = 0.02), with an absolute growth-rate of half of the no-HRT group (p = 0.02). Radiographic and clinical progression-free survival were 1.2 years and 3.3 years longer in the e-HRT group, respectively. These preliminary results suggest that e-HRT may be safe in incidental meningiomas.

## Introduction

Meningiomas are the most common primary intracranial neoplasm, accounting for more than half of all benign tumors of the central nervous system^1^. According to CBTRUS estimates, nearly 30,000 meningiomas are discovered annually in the United States. The diagnosis is usually incidental, and since these tumors are typically indolent, most require no immediate intervention. By definition, incidental meningiomas are usually small and not associated with neurologic symptoms at the time of discovery. Nonetheless, their growth over time may lead to significant morbidity and mortality. Some incidental meningiomas will eventually require treatment, which typically consists of surgery or radiotherapy. Meningiomas are two-to-three-fold more common in women compared to men. Their incidence shows a sharp rise in the 4th decade of life overlapping with the perimenopausal period in women^1–3^. Consequently, it is common for women diagnosed with meningiomas to be concurrently receiving or considering initiation of hormone replacement therapy (HRT).

Most meningiomas are known to express progesterone receptors (PR) and are often positive for estrogen receptors (ER) as well^4–8^. Unlike progesterone, estradiol was implicated as a potent enhancer of meningioma cell proliferation in vitro^4,9^, and ER-positive meningiomas were associated with a higher proliferative index than their ER-negative counterparts^7^. Therefore, there is a theoretical concern for accelerated tumor growth in women receiving estrogen HRT (e-HRT). This notion seems to be supported by a number of epidemiology studies that found increased meningioma incidence in women who received e-HRT^3,10,11^. Nonetheless, epidemiologic data on incidental meningiomas provide little guidance as to whether closer monitoring or a more aggressive approach to treatment is warranted if there is a history of prior HRT, or whether it is safe for a patient with these tumors to start or continue HRT. Unfortunately, in the absence of studies that assess the association of HRT with in vivo meningioma growth rate and clinical outcomes, clinicians have to rely on anecdotal evidence and expert opinion on how to manage HRT and incidental meningiomas concurrently^12^.

The present study aimed to provide clinically translatable evidence through a retrospective comparison of tumor growth patterns and clinical outcomes in meningioma patients who received estrogen hormone replacement therapy (e-HRT), as compared to those without a history of female sex hormone replacement therapy (no-HRT).

## Methods

We conducted a single-center retrospective case–control study of female patients monitored for probable meningiomas within the NorthShore University HealthSystem Neurology and Neuro-Oncology practice. We included women with radiographic findings consistent with an incidentally found (i.e., asymptomatic) meningioma and with six or more documented months of clinical and radiographic follow-up.

**Figure 1:**
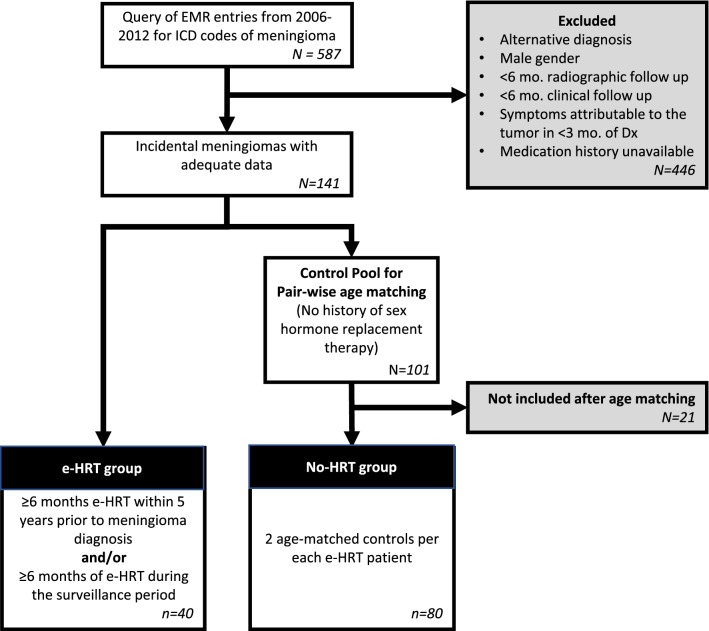
Selection flow chart.

All eligible patients were 18 years of age or older and were treated with e-HRT for at least 6 months, initiated within 5 years before or during their meningioma surveillance period. Treatment status was determined based on retrospective review of medication records and clinic notes. We compared each e-HRT patient with two age-matched female controls with a diagnosis of incidental meningioma, and no history of HRT, all of which were recruited from the same meningioma cohort (Fig. 1). This study was approved by the NorthShore University HealthSystem Institutional Review Board and all methods were carried out in accordance with relevant guidelines and regulations. The Informed Consent procedure was waived by the Northshore HealthSystem Institutional Review Board, based on retrospective nature of the study and no more than minimal risk to the subjects.

### Evaluation of growth rate

Baseline radiographic characteristics on MRI were compared between the two groups based on previously established parameters predictive of meningioma behavior, such as intensity on T_2_-weighted MRI, presence of calcification on CT, or on susceptibility-weighted MRI sequences, and the presence of perilesional edema^13,14^. Size measurements were performed using the contrast-enhancing regions on MRI, whereas calcification was assessed using susceptibility-weighted MRI, or CT scans acquired within a year from the initial MRI. We established size as the (a) single largest perpendicular dimension (1D) and (b) the largest biperpendicular diameter as described by McDonald et al. (2D)^15^. For both 1D and 2D measures, absolute annual growth rates (AGR: [final size − initial size]/follow up time in years) and proportional annual growth rates (PGR: AGR/initial size * 100%) were estimated. Growth trends were visualized on spaghetti plots and were qualitatively analyzed to identify differences in growth patterns. Meningioma size at diagnosis and end of follow-up, as well as growth rates, were compared between the e-HRT and no-HRT groups. Group comparisons for nominal parameters were performed using Fisher’s exact test. Continuous variables were first tested for normality; those with a normal distribution were compared using Student’s independent sample two-tailed T-tests, whereas non-normal variables were compared using the Mann–Whitney U-test.

### Evaluation of outcomes

Progression-free survival (PFS) was assessed radiographically, and defined by: (a) ≥ 5 mm AND ≥ 20% 1D increase as compared to the initial scan (RECIST 1.1)^16^; (b) ≥ 25% increase in 2D size compared to the first scan (and ≥ 3 mm increase in largest diameter if largest biperpendicular diameters < 10 mm at diagnosis, following RANO meningioma criteria)^15,17^; (c) time to clinical progression, defined as development of symptoms attributable to the meningioma or initiation of radiotherapy or resection. If multiple meningiomas were present, only the dominant (i.e., largest) lesion was measured*.* PFS group differences were assessed via the log-rank test and visualized using Kaplan–Meier curves. Cox regression analyses were performed to assess the relationship between PFS (radiographic and clinical) and the duration of hormone therapy, timing relative to diagnosis, HRT route, and BMI.

We repeated the same analyses for the following subgroups: (1) e-HRT via oral/subcutaneous/transdermal route only, (2) vaginal estrogen only, (3) e-HRT for at least one year before diagnosis, and (4) e-HRT for at least one year after diagnosis. The threshold for statistical significance was *p* < 0.05. Statistical analysis was carried out using IBM SPSS 25.0 (Armonk, NY).

## Results

### Patient characteristics

Forty e-HRT patients met all the eligibility criteria; 11 completed e-HRT before the meningioma diagnosis, 11 started e-HRT before and continued during the surveillance period, and 18 patients started e-HRT during surveillance (Table 1). Their mean age was 62.1 years (± 1.9). The mean duration of e-HRT was 5.0 years (± 0.7). Most patients (62.5%) received oral or transdermal products, whereas 30% used vaginal e-HRT, and 7.5% received e-HRT through more than one route. The age-matched control group consisted of 80 patients. Age at the end of follow up was similar between the groups, despite the e-HRT group had a non-significant trend toward having longer follow up (5.3 ± 0.4 vs. 6.8 ± 0.6 years, *p* = 0.061). Average BMI was significantly lower in the e-HRT group (26.3 ± 0.9 vs. 29.1 ± 0.9 kg/m^2^; *p* = 0.043). There was no difference in parity between the groups. Sixty-three percent of the e-HRT group had a history of hysterectomy.

**Table 1:** Study population.

no-HRT	N	(%)	Mean	± SE	(min–max)	*p* value^‡^
**Age (years)**						
At diagnosis	40	(100)	62.1	1.9	(37.0–93.2)	0.98
At end of follow up	40	(100)	68.9	1.8	(47.2–95.6)	0.57
Follow up time (years)	40	(100)	6.8	0.6	(1.2–16.4)	0.061
**HRT duration (years)**	40	(100)	5.0	0.7	(0.5–22.0)	–
Started and finished before diagnosis	11	(27.5)	3.7	12.8	(1.0–13.4)	–
Started after diagnosis	18	(45)	3.2	7.3	(0.5–9.6)	–
Started before, finished after diagnosis	11	(27.5)	9.4	1.5	(1.4–22.0)	–
**Route**						
Oral/transdermal	25	(62.5)	–	–	–	–
Vaginal	12	(30)	–	–	–	–
Both	3	(7.5)	–	–	–	–
**Estrogen agent**						
Estradiol	25	(62.5)	–	–	–	–
Conjugated estrogens	11	(27.5)	–	–	–	–
Multiple, other	4	(10)	–	–	–	–
**Other characteristics**						
Body-Mass Index (kg/m^2^)	40	(100)	26.3	0.9	(18.0–47.0)	0.043*
Number of children (parity)	39	(97.5)	2.1	0.2	(0–6)	0.65
**Radiographic characteristics (qualitative)**						
Calcified	18	(50)	–	–	–	0.35
Perilesional edema	3	(7.5)	–	–	–	0.54
T2-hyperintense	15	(38.5)	–	–	–	0.30

no–HRT	N	(%)	Mean	± SE	(min–max)	
**Age (years)**						
At diagnosis	80	(100)	62.2	1.3	(36.7–96.4)	–
At end of follow up	80	(100)	67.5	1.5	(38.8–97.2)	–
Follow up time (years)	80	(100)	5.3	0.4	(0.6–14.4)	–
**Other characteristics**						
Body-Mass Index (kg/m^2^)	80	(100)	29.1	0.9	(19.6–57.3)	–
Number of children (parity)	56	(70)	2.0	0.2	(0–6)	–
**Radiographic characteristics (qualitative)**						
Calcified	37	(56.1)	–	–	–	–
Perilesional edema	5	(6.3)	–	–	–	–
T2-hyperintense	35	(43.8)	–	–	–	–

^‡^Comparison of the e-HRT and no-HRT groups; continuous variables showed a normal distribution and Student's independent-sample two-tailed T-tests were used; categorical variables were compared using Fisher’s exact test (1-sided).

*Significant.

### Radiographic characteristics

Radiographic appearance, including the presence of calcification, edema, or T2-weighted intensity, were also similar between the e-HRT and control group (*p* ≥ 0.3). Meningiomas in the e-HRT group were smaller at diagnosis (13.0 mm vs. 16.1 mm) and remained smaller at the end of follow-up (15.1 vs. 18.7 mm; *p* = 0.03 for both comparisons; Table 2). The 2D measurements revealed similar group differences. Although both absolute and proportional growth rates were 1.5–2.3 times higher in the no-HRT group across 1D and 2D measures, only the 2D AGR difference was statistically significant (28.0 mm^2^/year vs. 12.1 mm^2^/year; *p* = 0.023). Visualized growth patterns were similar between the two groups (Supplemental Fig. 1).

**Table 2:** Meningioma dimensions and growth rates.

Dimensions	Group	Mean	± SE	(min–max)	*p*
Largest diameter at diagnosis (1D)	e-HRT	13.0 mm	1.0	(4–29)	0.026
	No-HRT	16.1 mm	1.1	(5–56)	
Largest diameter at end of follow up (1D)	e-HRT	15.1 mm	1.1	(5–31)	0.034
	No-HRT	18.7 mm	1.2	(7–63)	
Largest biperpendicular diam. at diagnosis (2D)	e-HRT	177 mm^2^	26.7	(12–702)	0.032
	No-HRT	276 mm^2^	37.5	(15–1946)	
Largest biperpendicular diam. at end of follow up (2D)	e-HRT	241 mm^2^	34.1	(25–914)	0.024
	No-HRT	369 mm^2^	44.4	(42–2066)	
1D annual absolute growth rate (AGR)	e-HRT	0.36 mm/year	0.1	(− 0.8–3.3)	0.16
	No-HRT	0.74 mm/year	0.2	(− 0.7–12.5)	
1D annual proportional growth rate (PGR)	e-HRT	3.3%/year	1.0	(− 4.9–24.2)	0.20
	No-HRT	5.5%/year	1.4	(− 7.5–89.2)	
2D annual absolute growth rate (AGR)	e-HRT	12.1 mm^2^/year	3.6	(− 16.9–108.6)	0.023
	No-HRT	28.0 mm^2^/year	5.9	(− 27.1–279.5)	
2D annual proportional growth rate (PGR)	e-HRT	9.7%/year	2.6	(− 6.9–77.5)	0.26
	No-HRT	15.3%/year	3.2	(− 12.4–166.3)	

In the subgroup analysis, e-HRT and control groups maintained the same distribution of most averages, as noted in the whole group analysis, but none of the comparisons reached statistical significance.

### Outcome analysis

**Figure 2:**
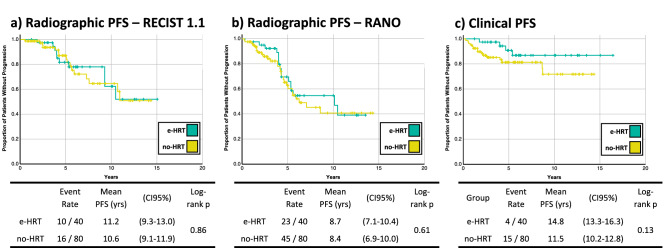
Kaplan–Meier curves of progression-free survival (PFS), based on (**a**) the largest diameter (mm; RECIST 1.1), (**b**) the largest biperpendicular diameters (mm^2^, RANO), or (**c**) clinical criteria where progression is defined as new symptom attributable to the meningioma, or initiation of treatment. For patients with multiple meningiomas, only the dominant lesion was included in the measurement. Vertical lines represent censored cases.

Mean PFS defined by RECIST 1.1, RANO, and clinical criteria were 11.2, 8.7, and 14.8 years in the e-HRT group versus 10.6, 8.4, and 11.5 years in the control group, respectively (Fig. 2). None of the differences were statistically significant (log-rank *p* > 0.1 in all analyses). Duration of e-HRT did not have an effect on either radiographic or clinical progression-free survival in the e-HRT group (HR = 1.00, *p* > 0.2; Supplemental Table 2).

The subgroup analyses showed consistently lower risk of progression in the e-HRT group, but none of these comparisons reached the level a statistical significance (Supplemental Table 2).

## Discussion

In this retrospective study of incidental meningiomas, we found consistently smaller tumors in those receiving estrogen hormone replacement within five years of discovery of meningioma or during surveillance. The differences were most pronounced on 2D measurements, revealing 35% smaller tumors in the HRT group both at diagnosis and the at end of follow-up. Growth rates were about twofold slower in the hormone replacement group, but only the difference in absolute 2D growth rates was significant in this cohort. Clinical PFS was 3.3 years longer for the e-HRT group, whereas radiographic PFS was very similar to the no-HRT group. No additional subgroup differences were revealed based on HRT route, timing relative to diagnosis, duration of the regimen used.

Our findings add clinical nuance to the limited and often conflicting evidence regarding the effect of e-HRT on meningiomas and their growth-rates. On a cellular level, PR expression is nearly ubiquitous in grade I meningiomas. In contrast, the rate of ER positivity has been reported in a wide range from 1 to 48%, which is likely a result of variability in measurement methods, and is possibly underestimated due to the preoperative use of glucocorticoids that can inhibit sex hormone receptor expression^4,6–8,18^. Of note, ER expression has been associated with higher meningioma proliferative rates in some in vitro and ex vivo studies^7,9^. Yet, on the individual level, *in human* data is restricted to case series of meningioma progression during pregnancy and extrapolation of experience with other cancers, such as the higher rate of progression in previously treated breast cancer patients^19–22^. On a population level, epidemiology studies that assessed the overall risk of meningioma development in women using HRT also yielded inconsistent results^11,23–27^. One of the most extensive case–control studies involved over 1000 cases and controls and concluded no statistically significant risk for meningioma development with HRT^23^. In contrast, a meta-analysis found the pooled odds ratio to be 1.29 compared to the HRT naïve^11^. A more recent case–control study looking at an ethnically diverse population found an odds ratio of 1.09 for e-HRT^27^. However, to the best of our knowledge, none of these studies have reported meningioma growth-rates or any form of outcomes in patients receiving e-HRT.

The majority of meningiomas are discovered incidentally and have a slow growth-rate. Most only require surgical intervention years or decades after the initial diagnosis, if any at all. Given these intrinsic tumor characteristics, studies assessing outcomes in meningiomas are unable to use overall survival as an appropriate outcome measure, and radiographic outcome measures are still under development^17^*.* In our study, we assessed radiographic (1D and 2D) as well as clinical PFS as surrogate measures for tumor behavior. Although semi-automated 3D volumetric analysis may be more sensitive to detect subtle tumor growth and can be especially useful for monitoring en plaque meningiomas in a research setting, it requires advanced image processing that is not widely available in the clinical practice, neither is it standardized^17^. Instead, the present study is intended to emulate information gathering in the day-to-day clinical setting. 1D and 2D measurements, as well as clinical data, can be quickly performed in the clinical setting. Some may argue that the association between growth rate and HRT exposure may only be evident after a long treatment period, given the slow progression of these tumors. However, a recent study of incidental meningiomas found a plateauing of growth-rate in these tumors after five years. ^14^ Thus, the almost seven years of follow up and five years of e-HRT should be sufficient to reveal significant growth acceleration. Both the statistically significant and non-significant differences found in the present study may suggest the opposite: on average, patients in the e-HRT group completed their follow up with smaller meningiomas, as opposed to controls. Therefore, there is no clear trend to indicate a possible masked effect due to short follow-up time. Also, the duration of hormone replacement was not a significant risk factor for radiographic or clinical progression in the main group nor the subgroup analysis. Lastly, it is unclear whether this group difference is due to chance due to the lower BMI in the e-HRT group. Higher BMI had been previously connected to a higher risk to develop meningiomas^23,27^.

### Limitations

The most significant limitations of this study are due to the relatively low sample size and the retrospective design. The latter evokes the potential risk of differential reporting on medication use in cases vs. controls. In order to improve the accuracy of data collection, our study selected patients based on records of medication prescription, as opposed to reported use.

Another limitation of our study is the non-uniform timing of e-HRT in relation to diagnosis and monitoring of the meningiomas; 28% of patients in this cohort had already completed e-HRT by the time of their meningioma diagnosis. Measuring the diameters on the last MRI available should still allow size comparison as it designates the end of exposure for all the patients included.

This analysis was performed in a progestin agnostic fashion as the majority of e-HRT patients had a history of hysterectomy, and thus the low number of patients receiving progestins was insufficient for a meaningful analysis.

Lastly, the focus on incidental meningiomas may have introduced selection bias through automatic exclusion of cases with a symptomatic presentation. Among these could theoretically be a group of estrogen “super sensitive” tumors with a highly accelerated growth-rate after e-HRT is started. However, the existence of such group is not supported by our data from patients who started e-HRT during the surveillance period.

## Conclusion

In conclusion, this is the first study to our knowledge that reports on growth-rate of meningiomas in patients receiving estrogen HRT. We found a similar to lower meningioma growth-rate in women exposed to estrogen HRT during the surveillance period, and up to 5-years prior to diagnosis compared to women not receiving estrogen HRT. Our study has the potential for changing practice in suggesting it may be safe for women to continue estrogen-based HRT after diagnosis of a meningioma; however, the role of progestins in HRT is not addressed here. Additionally, our study suggests that women with meningiomas taking estrogen HRT do not require more aggressive monitoring than other patients. A larger prospective case–control study is necessary provide further support for our findings.

## Supplementary information

Supplementary Figure 1.

Supplementary Table 1.

Supplementary Table 2.
