# Supplementary material for: Estrogen hormone replacement therapy in incidental intracranial meningioma: a growth-rate analysis
Source: Sci Rep. 2020 Oct 21;10:17960. doi: 10.1038/s41598-020-74344-x (PMC7578640; doi:10.1038/s41598-020-74344-x)
Supplement: Supplementary file 1 — Supplementary Figure 1. [file 41598_2020_74344_MOESM1_ESM.pptx]

## Slide 1
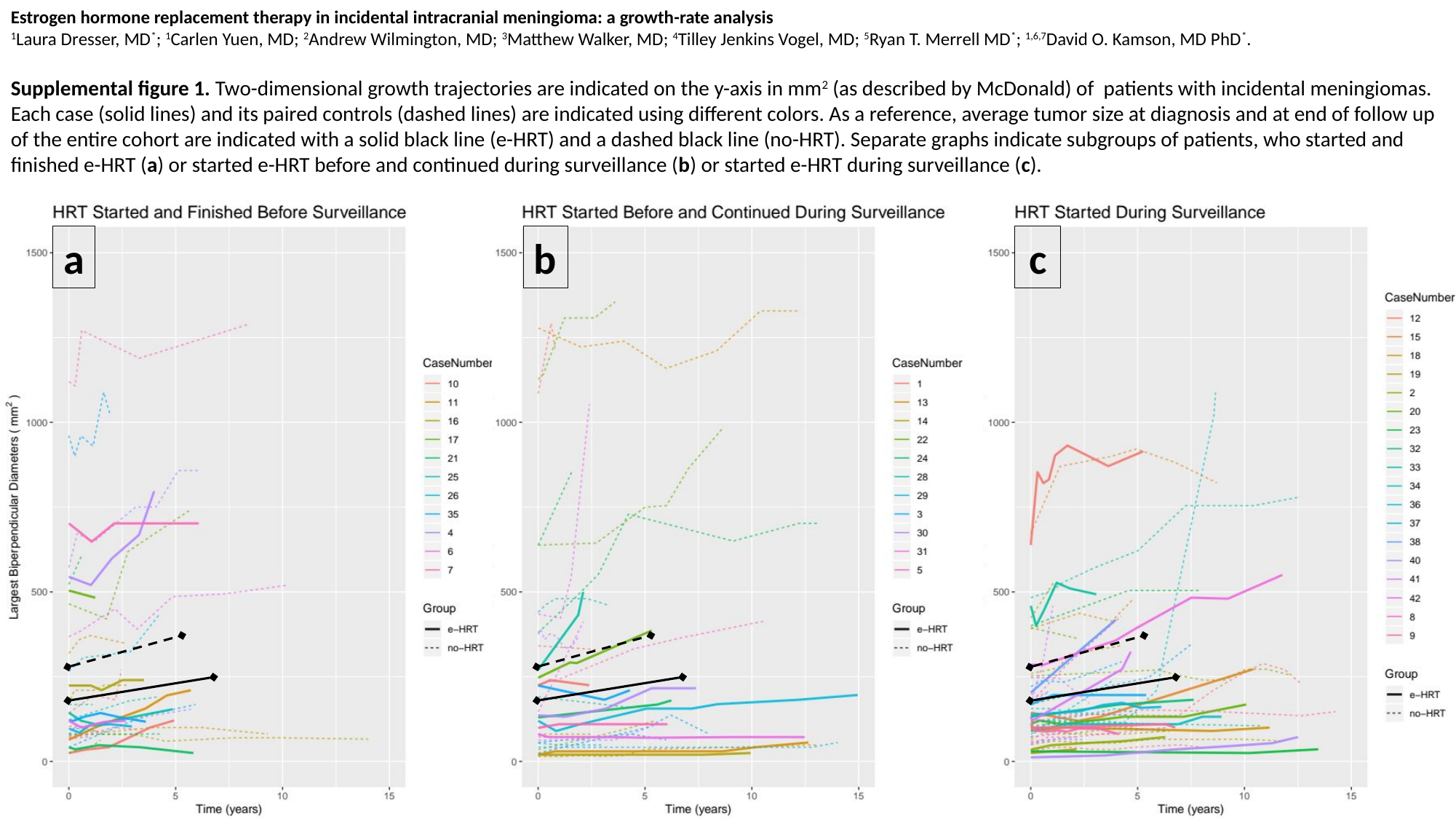

Estrogen hormone replacement therapy in incidental intracranial meningioma: a growth-rate analysis
1Laura Dresser, MD*; 1Carlen Yuen, MD; 2Andrew Wilmington, MD; 3Matthew Walker, MD; 4Tilley Jenkins Vogel, MD; 5Ryan T. Merrell MD*; 1,6,7David O. Kamson, MD PhD*.
Supplemental figure 1. Two-dimensional growth trajectories are indicated on the y-axis in mm2 (as described by McDonald) of patients with incidental meningiomas. Each case (solid lines) and its paired controls (dashed lines) are indicated using different colors. As a reference, average tumor size at diagnosis and at end of follow up of the entire cohort are indicated with a solid black line (e-HRT) and a dashed black line (no-HRT). Separate graphs indicate subgroups of patients, who started and finished e-HRT (a) or started e-HRT before and continued during surveillance (b) or started e-HRT during surveillance (c).
a
b
c
